# Collagen Fibrils in Skin Orient in the Direction of Applied Uniaxial Load in Proportion to Stress while Exhibiting Differential Strains around Hair Follicles

**DOI:** 10.3390/ma8041841

**Published:** 2015-04-20

**Authors:** Sterling Nesbitt, Wentzell Scott, James Macione, Shiva Kotha

**Affiliations:** Biomedical Engineering, Rensselaer Polytechnic Institute, Troy, NY 12180, USA; E-Mails: sterling.nesbitt@gmail.com (S.N.); wentzs@rpi.edu (W.S.); james.macione@gmail.com (J.M.)

**Keywords:** second harmonic generation microscopy (SHG), fast fourier transform (FFT), collagen orientation index, digital image correlation (DIC), strain mapping

## Abstract

We determined inhomogeneity of strains around discontinuities as well as changes in orientation of collagen fibrils under applied load in skin. Second Harmonic Generation (SHG) images of collagen fibrils were obtained at different strain magnitudes. Changes in collagen orientation were analyzed using Fast Fourier Transforms (FFT) while strain inhomogeneity was determined at different distances from hair follicles using Digital Image Correlation (DIC). A parameter, defined as the Collagen Orientation Index (COI), is introduced that accounts for the increasingly ellipsoidal nature of the FFT amplitude images upon loading. We show that the COI demonstrates two distinct mechanical regimes, one at low strains (0%, 2.5%, 5% strain) in which randomly oriented collagen fibrils align in the direction of applied deformation. In the second regime, beginning at 5% strain, collagen fibrils elongate in response to applied deformation. Furthermore, the COI is also found to be linearly correlated with the applied stress indicating that collagen fibrils orient to take the applied load. DIC results indicated that major principal strains were found to increase with increased load at all locations. In contrast, minimum principal strain was dependent on distance from hair follicles. These findings are significant because global and local changes in collagen deformations are expected to be changed by disease, and could affect stem cell populations surrounding hair follicles, including mesenchymal stem cells within the outer root sheath.

## 1. Introduction

**Figure 1:**
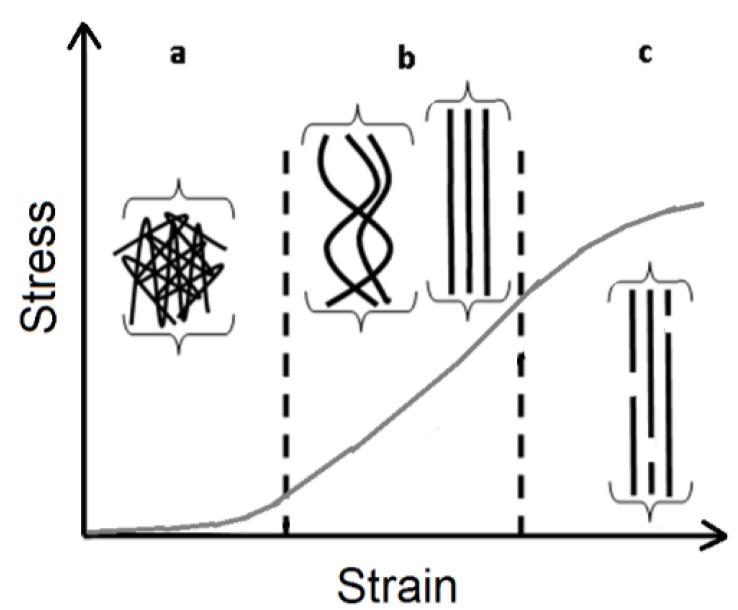
The stress-strain relationship of the skin is associated with collagen orientation. (**a**) The collagen fibrils are randomly aligned and readily orient with the application of small amounts of stress (low elastic modulus); (**b**) The collagen fibrils begin to straighten and gradually align in the direction of the applied stress. At the end, almost all collagen fibrils are aligned in the direction of loading; (**c**) Damage begins to accumulate due to sliding of fibrils past one other, among many other mechanisms. Figure adapted from Brinckmann [1] and Daly [3].

The biomechanics of skin is complex, typically demonstrating three deformation regimes in response to stress (Figure 1). Initially, the collagen is oriented in a random pattern (Figure 1a) [1]. While the collagen fibrils are in this random pattern, it orients readily to the applied load, hence demonstrating low elastic modulus [1,2]. Within this region, the elastic behavior of skin is not determined by collagen, but rather, an elastin mesh [1]. As the skin is stretched further, the collagen fibrils straighten and realign parallel to one another, thus, requiring more load to induce further elongation (Figure 1b) [1,2,3]. This process can continue until the fibrils are mostly aligned in the direction of the applied load (Figure 1b). Subsequently, failure occurs as fibrils begin to slide past one other, or by a multitude of other mechanisms (Figure 1c).

To determine the mechanical behavior of skin, most studies have used the initial orientation of skin collagen fibrils. The initial orientations of the collagen fibrils were obtained by histological analysis of excised skin. Upon application of load, the orientation of collagen fibrils is assumed to change, leading to increased load bearing by collagen fibrils. However, the change in collagen fibril orientation under different stress magnitudes has yet to be characterized experimentally. Experimental characterization of collagen fibrillar orientation is now possible, because of advances in multiphoton microscopy and because collagen fibrils emit second harmonic generation (SHG) due to their inherent non-centrosymmetry [4]. Within the last few years, the periodic structure of collagen fibrils in skin has been analyzed using fast fourier transforms (FFT) of SHG images. The amplitude component in the FFT of SHG images indicates the frequency of spacing between collagen fibrils. This parameter has been demonstrated as a diagnostic potential for medical disorders, including, distinguishing between healthy and diseased dermis [5,6,7], evaluating skin cancer [8], accessing photoaging [9], and scarring [10], among a diverse array of other applications.

Mechanical loading may contribute towards skin biomechanical behavior by regulating stem cells residing around hair follicles, including mesenchymal stem cells in the outer root sheath [11,12]. Therefore, an understanding of the deformation of collagen fibrils around these discontinuities could lead to an understanding of how the skin cells regulate its collagen content, as a means to achieve a desired mechanical environment, as well as during tissue regeneration. Strains in and adjacent to discontinuities have been investigated using digital image correlation (DIC) [13,14]. DIC has been used to determine inhomogeneity of strains on the surface of soft [15,16] and inside hard tissues [17], but, strains in tissues with pigmentation, such as skin, have not been evaluated.

The goal of this paper is to determine the stress-collagen fibril orientation relationship by utilizing the symmetry of the FFT of SHG images. A further goal of this manuscript is to determine the inhomogeneity of strains, especially, as it relates to the presence of discontinuities, such as hair follicles. Since the deformation of collagen fibrils is primarily responsible for soft tissue deformation and subsequent mechanobiological response, and since collagen fibril orientation at various depths can be measured non-invasively, this has the potential to add to our understanding of its contribution in various disease states.

## 2. Results and Discussion

### 2.1. FFT Analysis of Global Deformation

**Figure 2:**
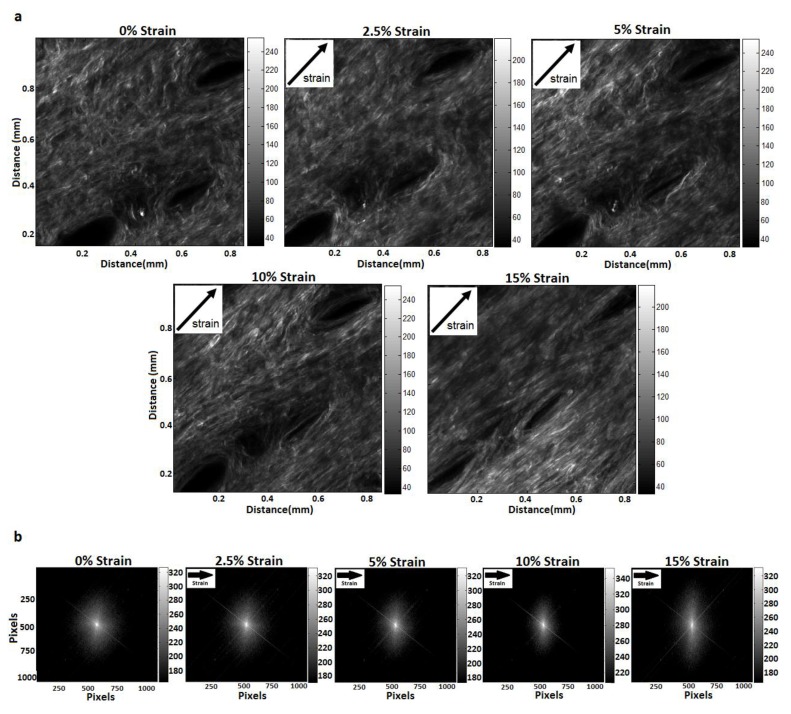
Representative Second Harmonic Generation (SHG) images and the respective fast Fourier transforms (FFT) for a random sample. (**a**) SHG images of skin at different strain magnitudes. Strain was applied at 45° to the x-y scans. Each image is single frame from a 70–100 z-stack images obtained at each strain level. As the strain increases, a greater number of fibrils align in that direction; (**b**) FFT amplitude images reoriented in the direction of applied strain (horizontal direction). Arrows indicate the direction of applied strain.

Skin stretching causes collagen fibrils to change orientation towards the direction of applied load (Figure 2a). SHG images of skin taken at low strain (0%, 2.5% strain) indicate that the collagen fibrils (white lines) appear to be coiled and wavy. At 5% and 10% applied strain, the collagen fibrils appear more organized along the axis in which the strain is applied and some of the waviness disappears. At the largest strain (15%), the collagen fibrils appear aligned along the axis of induced strain.

The shape of each FFT (Figure 2b) can be visually compared to the spatial frequency of the collagen fibrils (Figure 2a). Note that the arrows indicate the direction of applied load (direction of arrows is different in Figure 2a,b). As strain increases, the collagen fibrils orient along the direction of applied load and become more densely packed, *i.e.*, periodicity decreases, in the perpendicular direction. As a result, there is an increase in the higher frequency components in the FFT amplitude images perpendicular to the direction of applied load (Figure 2b). Thus, the FFT images appear to become more ellipsoidal, which is characterized by a parameter termed COI (Equation (2)–see Methods).

**Figure 3:**
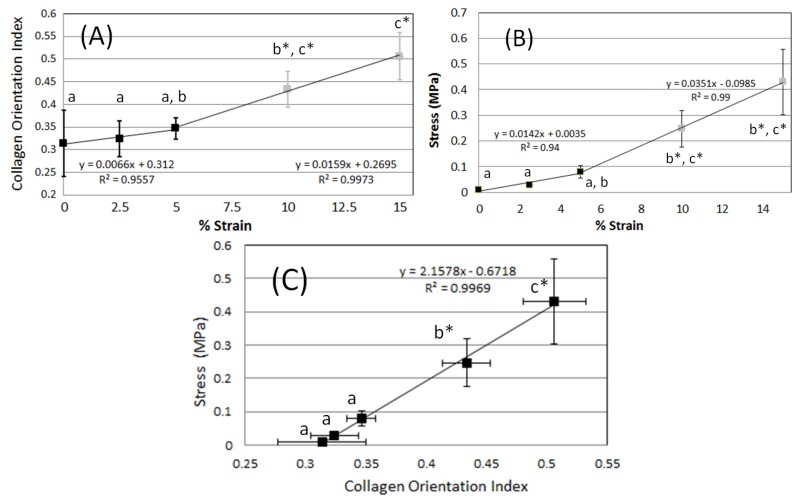
The collagen orientation index (COI) and stress plotted as functions of induced strain. (**a**) The COI plotted as a function of induced strain. Linear regression is performed on two different regions above and below 5% strain; (**b**) Stress *vs.* strain demonstrates similar properties to COI *vs.* strain; (**c**) Stress (used to induce strain) *vs.* COI demonstrates high correlation. Error bars indicate standard deviation and *n =* 4. Similar letters indicate that the groups are statistically similar. The symbol * indicates power > 0.8.

Applied strains, stresses and COI were compared to one other. ANOVA followed by Tukey *post-hoc* analysis, was performed using R (R 3.01, http://www.r-project.org/). Analysis of COI *vs.* strain relationships (Figure 3a) indicated that the COI’s at 0% and 2.5% are different from COI’s at 10% and 15% (*p <* 0.05 and power > 0.8). Also, the COI at 5% were found to be different from COI’s at 15% (*p <* 0.05 and power > 0.8). The COI is observed to be non-linear with respect to applied strain, with two distinct regimes observed above and below the 5% strain threshold, that were fit linearly (Figure 3a). At less than 5% strain, there is a slope of approximately 0.0066 (first regime, *R^2^* = 0.9557), which, at greater than 5% strain, increases to 0.0159 (second regime, *R^2^* = 0.9973), an increase of 2.4 times (*p <* 0.05, power > 0.8). Analysis of the stress *vs.* strain relationship (Figure 3b) indicated that stresses at 0% and 2% were different from stresses at 10% and 15%. The stress is also observed to be non-linear with respect to strain (Figure 3b), exhibiting two distinct regimes, occurring below and above 5% strain. A linear regression performed on both regions, *R^2^* = 0.9438 and 0.9994 below and above 5% strain, respectively; indicates that the slopes increases by 2.5 times from 0.0142 to 0.0351 (*p <* 0.05, power > 0.8). Analysis of the COI *vs.* stress relationship (Figure 3c) indicated that the COI’s at low stresses (that correspond to strains of 0%, 2.5% and 5%) were different from COI’s at higher stresses (that correspond to strains of 10% and the 15%) (*p <* 0.05, power > 0.8). Moreover, COI’s at stress that corresponds to 10% strain was different from the stress that corresponds to 15% strain (*p <* 0.05, power > 0.8). In contrast to the non-linear relationship observed between stress *vs.* strain and COI *vs.* strain, the COI and applied stress relationship (Figure 3c) demonstrates a strong linear relationship between the orientation of collagen and the strain being applied to the skin (*R^2^* = 0.9969).

### 2.2. DIC Analysis of Local Deformation by Strain Maps

**Figure 4:**
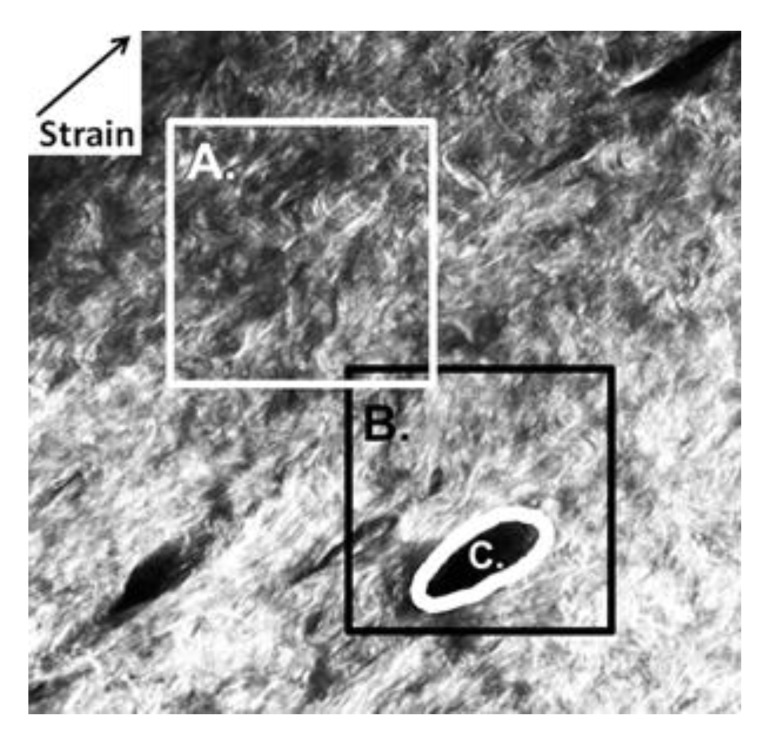
Maximum and minimal principal strains were averaged over three different regions; collagen matrix region (white box denoted by **A**), surrounding hair follicle collagen (black box denoted by **B**), and hair follicle collagen, (which is the area covered by the white ellipse **C**). The direction of applied deformation is indicated in the upper left hand corner.

To determine inhomogeneity of local strains, strains in three regions were investigated based on distance from the hair follicle, namely, region with primarily collagenous matrix, region surrounding the hair follicle, and a thin region immediately adjacent to the hair follicle (labeled as regions A, B, and C in Figure 4).

**Figure 5:**
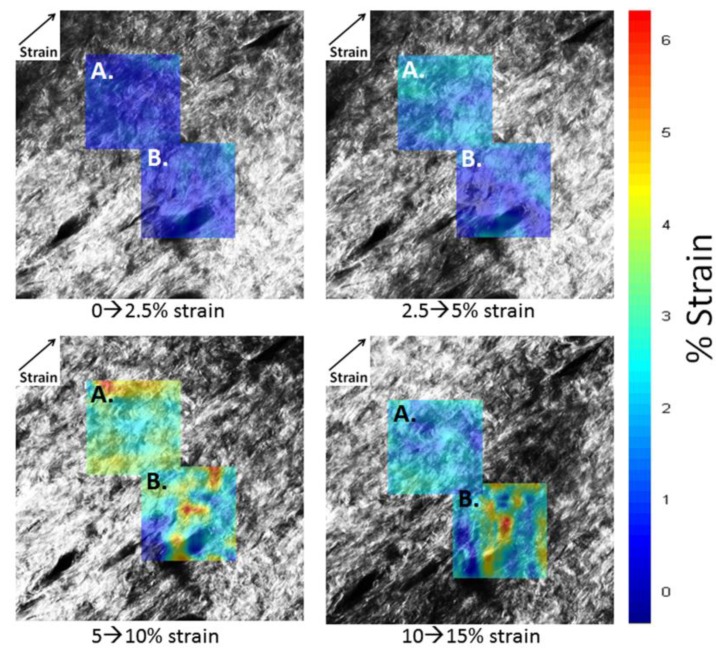
Maximum principal strain maps for matrix region (**A**) and hair follicle region (**B**). Percent strain is given as the difference between images as indicated. Strain direction is indicated in the upper left corner.

**Figure 6:**
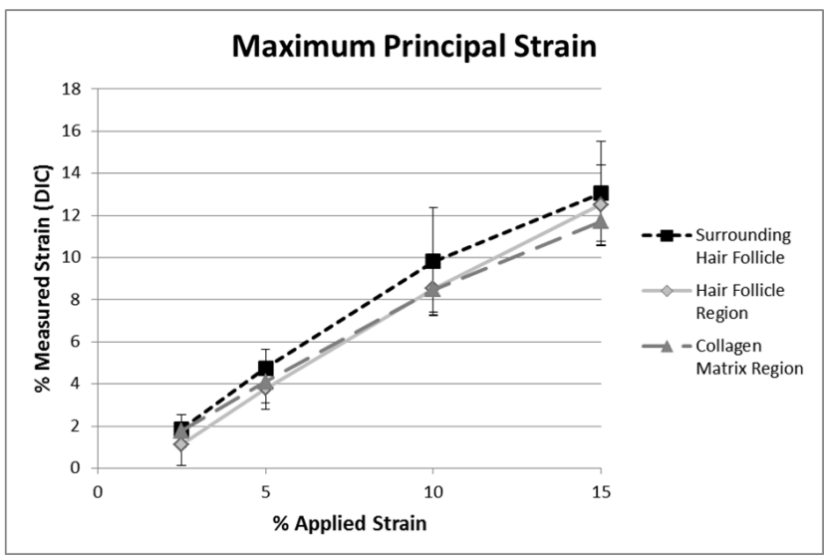
Maximum principal strains averaged over the three regions indicated 4, *versus* applied strain. All three regions of collagen (*i.e.*, hair follicle, surrounding hair follicle, and matrix) increase with applied strain (*p* < 0.001). There is no statistical significance between locations. Error bars represent ±1 Standard deviation.

Maximum principal strain was found to be positive over all collagen regions (Figure 5 and supplementary Figure S1). Figure 5 shows maximum principal strains scaled to the color map on the right for percent strain. The color map is then overlaid onto gray scale SHG images to show underlying structure of collagen fibrils as a function of stretch. Averaged strain values (Figure 6) are lower than the overall applied strain, which was determined from the separation between grips as measured using a Vernier caliper. This is expected as there are typically higher strain values immediately around the grips, making strain levels in the center of the sample lower. It was found that maximum principal strain increased for all three areas (collagen matrix, collagen surrounding the hair follicle, and hair follicle) with respect to increasing applied strain, as determined by linear regression (*p* < 0.001). Results from ANOVA indicated that there was also no interaction between applied strain and location (*p* = 0.93).

There was some non-linearity in averaged strains in the 10%–15% strain regime. This difference may be because of relative slippage of fibrils in skin as it was stretched beyond 10% strains, as observed in the change in direction of principal strains at some locations (Figure S1, bottom left).

**Figure 7:**
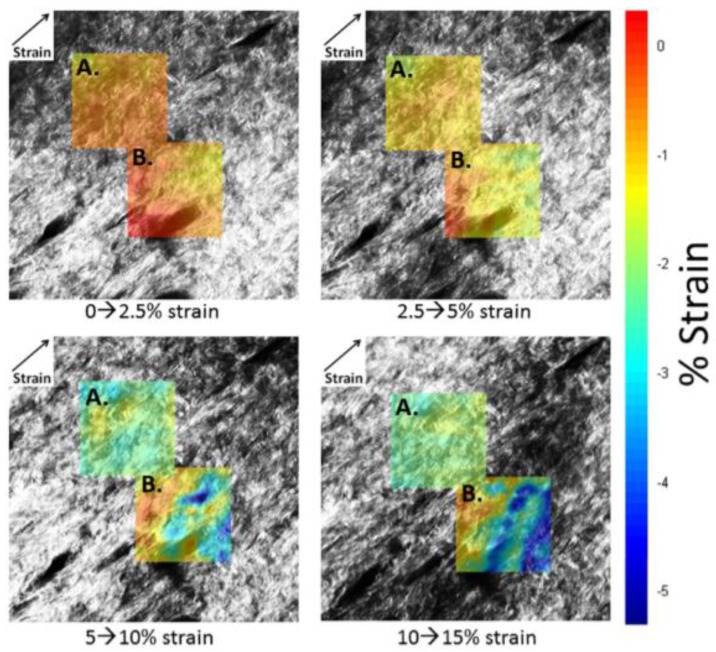
Minimum principal strain maps for matrix region (**A**) and hair follicle region (**B**). Percent strain is given as the difference between images as indicated. Strain direction is indicated in the upper left corner.

**Figure 8:**
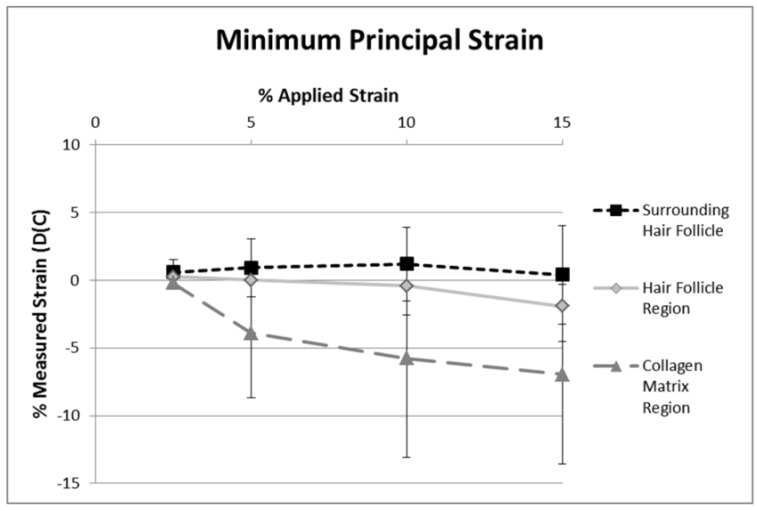
Averaged minimum principal strain, as measured by DIC, *versus* applied strain. There was a statistically significant difference (*p* = 0.02) between the different locations. Minimum principal strains immediately surrounding the hair follicle were positive, indicating an expansion of collagen in this zone. In contrast, minimum principal strains in primarily collagenous zones were negative, indicating a Poisson’s effect. Error bars represent ±1 Standard deviation.

Minimum principal strains were generally negative (Figure 7), as would be expected because of the Poisson’s ratio effect, perpendicular to the direction of stretch. In contrast to maximum principal strains, minimum principal strains were affected by the collagen’s location, *i.e.*, proximity with respect to hair follicles (*p* = 0.02). Minimum principal strains in predominantly collagen rich regions (Figure 8) consistently become more negative with higher applied strains of up to 5%, and only gradually increased thereafter (Figure 8). Averaged minimum principal strains determined from collagen fibril deformation surrounding the hair follicle were slightly negative, whereas, they were slightly positive in the hair follicle region. This indicates that the hair follicles are expanding in the direction perpendicular to applied strain. Minimum principal strain was shown to only be significantly different between the hair follicle and collagen matrix regions (*p <* 0.01), and moderately different between surrounding hair follicle and collagen matrix regions (*p =* 0.10). Furthermore, minimum principal strains at all three locations were independent of applied strain (*p* = 0.60).

### 2.3. Analysis and Limitations

Reorientation of collagen fibrils was assessed by FFT of SHG images under different strain magnitudes (global changes) while the inhomogeneity of strains were determined from DIC of SHG images (local changes). SHG images were obtained after complete stress relaxation had occurred. As the skin is stretched, collagen fibrils re-orient towards the direction of applied strain (Figure 2a). This change in fibril orientation leads to a change in the shape of the FFT (Figure 2b) which is quantified with the COI (Figure 3a). The COI demonstrates two regions of skin stretching, when the collagen starts to reorient (0%, 2.5%, and 5% strain) and when the collagen fibrils start to elongate (5%, 10%, and 15% strain). These two regions compare well with the known stress-strain analysis of skin (Figure 1) and also with the measured stress-strain relationship (Figure 3b). In contrast to the two linear regions observed for the COI-strain and stress-strain relationships, there is only one linear relationship for the stress *vs.* COI for the strain ranges investigated (Figure 3c). This indicates that the proportion of collagen fibrils re-orienting is proportional to the applied load.

DIC of SHG images allowed us to measure the maximum and minimum principal strain based on deformation of collagen fibrils, especially in proximity to discontinuities such as hair follicles. It was found that maximum principal strain was highly affected by applied uniaxial strain and was unchanged by the presence of discontinuities. Minimum principal strain was affected by the location of the collagen matrix with respect to the discontinuity (*i.e.*, the hair follicle). Minimum principal strain in the predominantly collagen matrix region (region A in Figure 4) demonstrated that the collagen fibrils are closer in the direction perpendicular to direction in which it was stretched (Poisson’s effect). However, collagen surrounding hair follicles exhibited slightly negative minimum principal strains and collagen in the hair follicle region has a positive minimal principal strain, indicating it is expanding in the direction perpendicular to the direction of stretching. This has important implications for the mechanobiological response of skin, because of the presence of stem cells in the hair follicle region.

Most studies of tissues that have utilized SHG to evaluate changes in organization to collagen fibrils have evaluated damage or changes to the collagen fibrils after applying loading of different types, typically, after inducing some type of damage—not, when the tissue is being subjected to mechanical loading [18,19,20,21]. Recent studies have begun to characterize changes in orientations of bundles of collagen fibrils in soft tissues (e.g., heart valves) in response to external load, by measuring 3-D changes in angle of bundles of collagen fibrils using a fit to the FFT of the SHG images [22]. One angle was used to characterize the averaged orientation of the fibrils in a 2-D plane, which is equivalent to the use of the major axis of the ellipsoid, in this study. As this axis does not change orientation during loading, we used changes in the ratio of the two axes (defined as COI) to characterize soft tissue mechanical behavior. It is noted that there are minimal confounding effects when studying the heart valve (e.g., there is no hair, or pigmentation).

In complex tissue such as skin, experimental studies of skin deformation are mostly limited to measurements of homogenized deformation measurements on skin surfaces [23] or to evaluation of deformation after removing layers [24]. It is noted that studies have been performed using small-angle x-ray scattering (SAXS) to relate collagen orientation to tear strength, but, not to changes in orientation while loading of skin [25,26]. As a result, the mechanical contribution of collagen fibrils in skin while it is undergoing loading has been mostly studied using computational and analytical models [27,28,29,30]. In these models, almost the entire load bearing capacity of skin was ascribed to the behavior of collagen fibrils under load. In these models, and as observed in our experiments, collagen fibrils change orientation at low loads and they are more densely packed at high loads (in the direction perpendicular to loading).

These studies are important because determining local and global changes to the main load bearing constituent of skin (*i.e.*, collagen fibrils), can provide an enhanced understanding of the mechanobiological responses of soft tissues [22], and for regeneration of soft tissues [31]. This technique could potentially provide earlier detection of disease or improve the robustness of the phenotypes detected using SHG techniques which can currently provide gross pathological changes, but, are relatively insensitive to subtle and progressive pathological changes to the dermis [5,6,7,9,32], for example, through changes in the slope of the COI-stress relationship or in the local strains around structures where stem cells may be present.

One major limitation of our study is that load bearing at the individual collagen fibril scale was not assessed. Complementary studies using polarization resolved SHG to evaluate anisotropy factor during loading [33] may enable one to relate changes in the network to those at the individual fibril. Another limitation of this experiment is that SHG was performed at 5 discrete strains levels and loading was limited to strains of 15%. The duration of imaging at each strain (30 min for stress relaxation, and 30 min to obtain 100 z-stacks) means that it took over 6 h (including microscope start-up time) to image one sample. These long time-periods precluded us from obtaining SHG images at intermediate strain values. Strain values were limited to 15% in order to ensure that damage induced movement of collagen fibrils were not occurring during loading (zone III in Figure 1). Movement of collagen fibrils during imaging causes motion artifacts that would have caused distortions in the FFT patterns (as was observed in some of the samples that were loaded in this study). Another limitation was that z-direction strains were not accounted for. We used 3-D FFT to determine the three axes of the ellipsoid, but, differences were not found in the third dimension. Note that the third axis was extremely truncated as there were only 30 pixels in that direction as opposed to 2048 × 2048 pixels in the *X-Y* axis. Furthermore, qualitatively, most of the fiber re-orientation was observed to take place along the long-axis. Another limitation was that the bi-axial behavior of skin was not measured, primarily because of the time required to conduct imaging. Another limitation is that images were obtained half an hour after stretching, a time-scale over which relaxation was complete, which would amplify the changes in orientation. Newer techniques, which can enable imaging of collagen fibril deformation quasi-static loading [34], might provide a more complete description of the physiological contribution of collagen fibrils to mechanical loading. A final limitation is that only a small region of the skin was assessed in few samples, and the results may not be representative.

## 3. Experimental Section

### 3.1. Sample Preparation

Skin was excised from nearly identical locations (the mid-central portion of the backs) of female, TOPGAL mice (roughly C57B6 background) of approximately 3 months of age. Before excision, the skin was shaved rigorously to remove hair, in order to remove spurious motion artifacts related to motion of hair, when stretching the skin. Each specimen was made into a strip that was approximately 10 mm × 50 mm and stored in tris-buffered saline (TBS) containing 0.05 wt% sodium azide. All experiments were performed within a week of excision. Individual specimens were about 2 mm thick, inclusive of the fat pad. Loading was performed in excised skin oriented along the cranial-caudal direction.

### 3.2. Imaging

Imaging was conducted using a Carl Zeiss LSM 5 multiphoton and confocal microscope in reflection mode consisting of a Zeiss Axioskop 2 FS upright scope, with a mode locked Ti:Sapphire laser (Chameleon Ultra II pulsed laser, Coherent Inc., Santa Clara, CA, USA) operating at 80 MHz and 0.7 W (20% of a full-scale average output power of 3.5 W). The incident laser wavelength was 800 nm, which was low-pass filtered at 680 nm, allowing second harmonics to pass. The reflected second harmonic was band-pass filtered (405/30 nm; Chroma) and intensity detected using an NDD PMT detector at 12-bit resolution and a gain setting of 346. Image stacks were obtained by scanning parallel to the skin surface using a Zeiss 20× water immersion objective (Zeiss W Plan Apo with 1.0 NA and WD of 1.8 mm). A stack of 70–100 images were obtained separated by 1 μm along the z-axis. Each image was 2048 × 2048 pixels, with a 0.5 µm/pixel resolution in the x-y plane. In these images, only a set of roughly 30 images contained SHG signal. The relevant 30 image stacks were isolated at each level of strain (0%, 2.5%, 5%, 10%, 15% strain) for 4 different samples. Note that multiple skin samples were imaged (*n =* 7) but, because of sample movement during an imaging period of 30 min per strain level, data at all strain values were obtained for only 4 samples (in 30 z-stack images) for FFT and in only 3 samples for DIC. So, only these samples were analyzed.

### 3.3. Stretching

**Figure 9:**
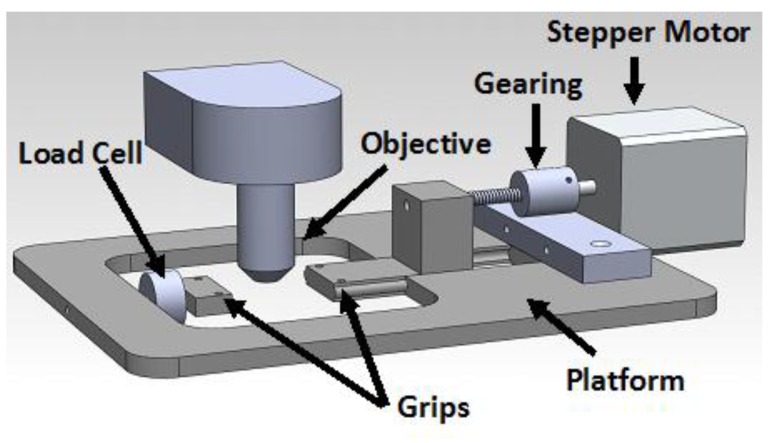
Schematic of a loading machine used to generate stress on the skin sample was made which allowed the skin to be stretched while under the objective of the multi-photon microscope.

In order to preserve the elastic properties of skin, it was kept wet using a tris-buffered saline drip throughout the imaging procedure. To generate controlled strain in the skin, the sample was placed between two grips which were below the microscope objective (Figure 9). Both ends of the grips were connected to a stepper motor through a gearing mechanism, which was able to generate displacements with a resolution of 0.5 µm. To insure that samples did not slip at the grips, grips were designed with pins and clamped. The initial distance between grips was 40 mm as measured by a caliper (0.01 mm resolution) and strain was applied to the samples by displacing the stepper motor through defined distances at a rate of 0.5 mm/min. After each level of strain was applied, the skin was given 30 min to relax. Note that if the collagen fibrils in the samples moved during an imaging period of 30 min, it was immediately discernible in the images. During this time, the stage was moved under the objective in order to obtain images from similar locations. Strains were applied in incremental order, starting with 0% and working to 15% strain (0%, 2.5%, 5%, 10%, 15%). The force generated was recorded with a 22.2 N load cell with a customized amplifier with ±0.05 N resolution [35]. The force at the end of 30 min of relaxation was used in stress calculations. Loads and displacements were recorded at 10 Hz. It is noted that the direction of loading was at 45° to the x-y scans performed by the servo motors on the objective as verified using images from the top of the sample stage.

### 3.4. Fourier Transform Power Spectra Analysis (FFT)

Each image stack (di(x,y)), which represents a set of 30 relevant images though the skin at a particular strain value, was imported into MATLAB for processing. Images were smoothed by filtering with a 9 × 9 array of ones to reduce high frequency noise in the FFT images. The 2-dimensional FFT of each image in the stack was shifted (fftshift) to move its low frequency components to the center of each image. The absolute value and then log of FFT was taken to reduce the dynamic range. The resultant sets of log compressed frequency transformed images were averaged into a two dimensional array, which can be expressed as the following:

(1) $$I\_FFT_{X,Y}=\frac{1}{n}\;\overset{n}{\underset{i=1}{\sum }}[{\text{log}}_{10}(abs|FFT2(d_{i}(x,y))|)]$$

**Figure 10:**
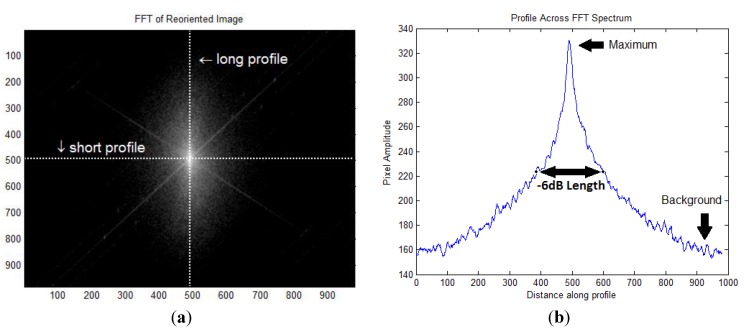
Measuring the short and long profiles (**a**) A FFT of an image of skin in which the lowest frequencies have been shifted to the center of the image. Two profiles (short and long) can be defined with respect to the induced strain; (**b**) When the short profile is examined, the FWHM (or –6 dB length) is a pixel distance between values which are half the amplitude between the maximum and background.

This averaging scheme is viable because the collagen fibrils in murine skin form a layered mesh network which oriented in the direction of applied load. As the x-y scans were performed at 45° to the loading axis, the FFT images were then reoriented by rotation to align with the axis of applied strain (the resulting FFT image is shown in Figure 10a). The symmetry of the frequency response (FFT) was evaluated by determining the length of two profiles across and normal to the axis of applied strain [7,9,10,36,37]. Different thresholds were chosen to determine the long- and short- axis of the ellipsoid in the FFT amplitude images. It is noted that results were not affected when using other thresholds, −3 dB to −8 dB values. Therefore, results are presented for the −6 dB value (which is full-width at half maximum or FWHM), as it is halfway between the maximum and background, in other words (maximum + background)/2 (Figure 10b). The FWHM was determined as the sum of pixels from the center of the image to the first point which was below the FWHM value (on each side) as shown in Figure 10b.

The FWHM lengths (short and long) across each of the dimensions would be used to compute the collagen orientation index (COI) [7,9,10,36]. The COI has been used to demonstrate the periodicity of collagen fibrils along and perpendicular to the direction of loading. The COI has been defined as,

COI = 1 − (short profile/long profile)
(2)

Thus, a tissue with random collagen orientation would have a COI of 0. As collagen micro-fibrils orient along the direction of applied load, they are squeezed in the perpendicular direction. In the FFT amplitude images, this change in periodicity is identified by increases in the frequency component perpendicular to the direction of applied load. Therefore, the long profile, which is perpendicular to the applied load, is expected to increase, resulting in increasing COI values. The short profile is expected to remain constant and serves as a normalizing parameter.

### 3.5. Digital Image Correlation (DIC)

DIC was performed to find maximum and minimum principal strain every twenty pixels apart and for 32 × 32 iterations along the x- and y-planes [17]. DIC created data sets consist of displacement (V and U), strain (VX and UY), and shear (VY and UX) values obtained every twenty pixels apart using a 100 × 100 pixel window. For each set of images, two DIC displacement data sets (32 × 32 iterations in the X- and Y- axis) were obtained based on proximity to hair follicles. The displacements, U and V, were corrected for rotation and filtered, following which they were interpolated to the size of the image (601 × 601 pixels). Strain values were determined using a Savitsky-Golay filter, from which maximum and minimum principal strains were calculated. Principal strain values were then averaged over three different areas of the image; the collagen matrix far away from hair follicles (601 × 601 pixels or 300 × 300 µm^2^ this conversion needs to be modified), the hair follicle (601 × 601 pixels), and the collagen immediately surrounding the hair follicle (10 pixels or 5 µm—adjacent to the hair follicle, where the collagen was found to be oriented along its perimeter) (Figure 6).

DIC was conducted between images that were close in applied strain (*i.e.*, between 0% and 2.5%, between 2.5% and 5%, *etc.*) resulting in 4 deformation maps for each sample. These averages were then summed such that all strain values are referenced from 0% strain (Equation (3)).

ε 0%→2.5% = ε 0%→2.5%
ε 0%→5% = ε 0%→2.5% + ε 2.5%→5%
ε 0%→10% = ε 0%→2.5% + ε 2.5%→5% + ε 5%→10%
ε 0%→15% = ε 0%→2.5% + ε 2.5%→5% + ε 5%→10% + ε 10%→15%
(3)

### 3.6. Statistics

DIC was performed to determine collagen fibril deformation adjacent to and far away from hair follicles. The images were divided into regions where hair follicles were absent (shown as white box in Figure 6), where hair follicles were present (shown as black box), and immediately next to the hair follicle (10 pixels along hair follicle perimeter–shown as white thick outline).

Two-way ANOVA was performed followed by Tukey’s significant difference test. Applied strain and the region of collagen were the independent variables, while averaged maximum and minimum principal strain values for skin collagen were the dependent variables. Mice were considered to come from the same population and were binned along with the dependent variables.

## 4. Conclusions

Analysis of SHG images of murine skin to discern local and global changes indicates that minimum principal strains are not-homogenous (especially being positive around hair follicles) as well as that closer packing of collagen fibrils perpendicular to the loading direction is correlated to stress. The global change is collagen fibril orientation with applied load is captured by the collagen orientation index (COI), which is a measure of the ratio of the FFT in the periodicity in the collagen fibrils along and perpendicular to the direction of applied load. The COI-strain and stress-strain relationships exhibit bi-linear behavior while the COI-stress relationship was linear over the range of strains studied. This data combined with previous computational models suggests that collagen fibrils in skin are primarily responsible in contributing to its mechanical behavior. Further studies are necessary to determine whether parameters, such as COI and strain inhomogeneity, can be used for sensitive detection of changes to the extracellular matrix as a consequence of disease.

## Supplementary Materials

Supplementary materials can be accessed at: http://www.mdpi.com/1996-1944/8/4/1841/s1.
