# Supplementary material for: Collagen Fibrils in Skin Orient in the Direction of Applied Uniaxial Load in Proportion to Stress while Exhibiting Differential Strains around Hair Follicles
Source: Materials (Basel). 2015 Apr 20;8(4):1841–57. doi: 10.3390/ma8041841 (PMC5507025; doi:10.3390/ma8041841)
Supplement: Supplementary file 1 [file materials-08-01841-s001.pdf]

Supplementary Materials

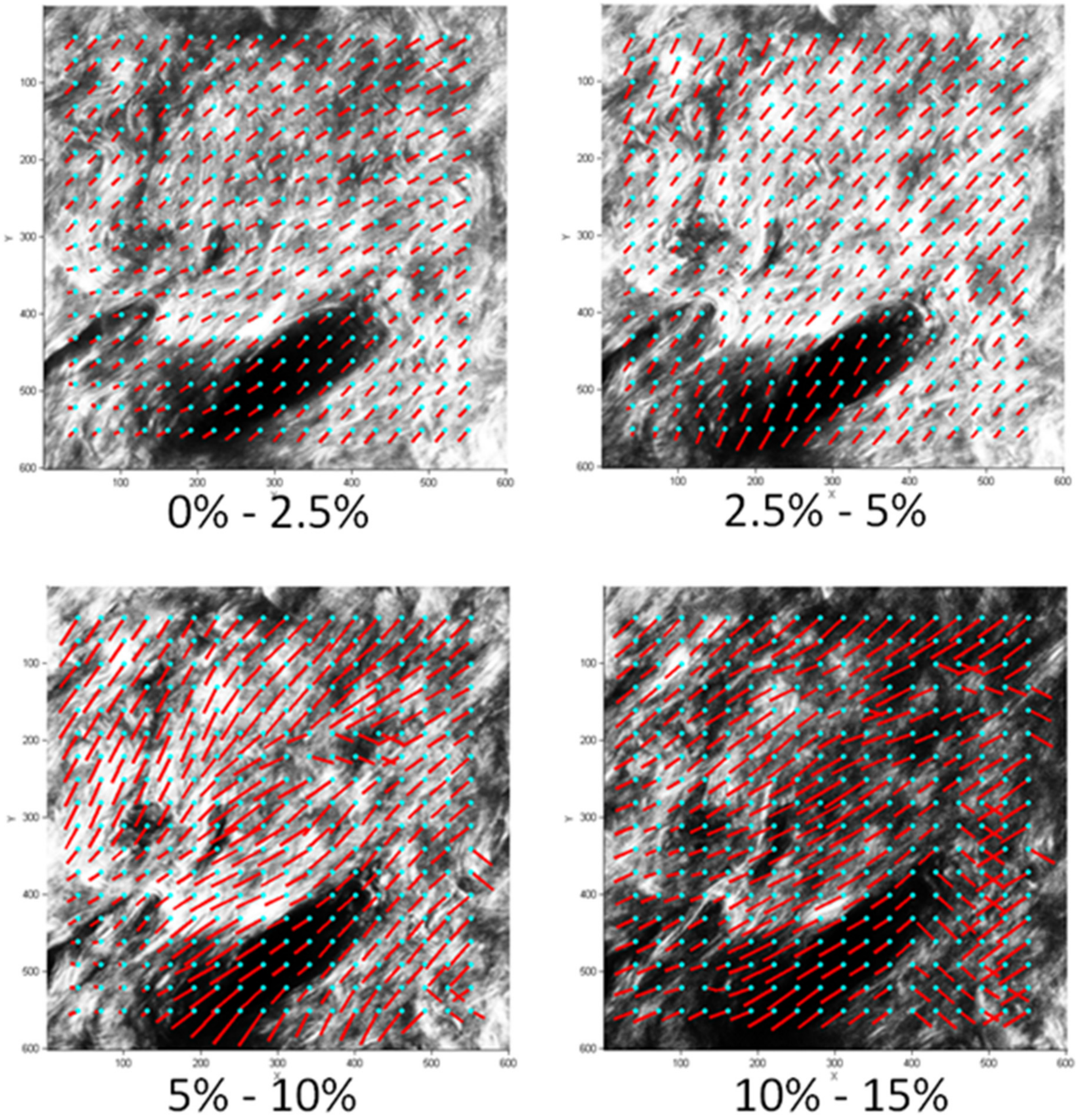

Figure S1. Directions and magnitude of maximum principal strains, shows the inhomogeneity of strains. In the figure, the origin is denoted in blue, and the direction in red. Most principal strains occur in the direction of applied deformation. However, in the 10%–15% strain regime, there are some sites that are not along the same axis. This may indicate sliding of the fibrils, and gives raise to the change in slope in the 10%–15% regime observed in Figure 6.
